# Multilevel attention mechanism for motion fatigue recognition based on sEMG and ACC signal fusion

**DOI:** 10.1371/journal.pone.0310035

**Published:** 2024-11-04

**Authors:** Dinghong Mu, Jian Wang, Fenglei Li, Wujin Hu, Rong Chen

**Affiliations:** 1 East China University of Technology, Nanchang, China; 2 East China Jiaotong University, Nanchang, China; Universita Politecnica delle Marche Facolta di Ingegneria, ITALY

## Abstract

This study aims to develop a cost-effective and reliable motion monitoring device capable of comprehensive fatigue analysis. It achieves this objective by integrating surface electromyography (sEMG) and accelerometer (ACC) signals through a feature fusion strategy. The study introduces a multi-level attention mechanism for classification, leveraging convolutional neural networks (CNNs). The preprocessing phase involves a local feature attention mechanism that enhances local waveform features using the amplitude envelope. A dual-scale attention mechanism, operating at both channel and neuron levels, is employed to enhance the model’s learning from high-dimensional fused data, improving feature extraction and generalization. The local feature attention mechanism significantly improves the model’s classification accuracy and convergence, as demonstrated in ablation experiments. The model, optimized with multi-level attention mechanisms, excels in accuracy and generalization, particularly in handling data with pseudo-artifacts. Computational analysis indicates that the proposed optimization algorithm has minimal impact on CNN’s training and testing times. The study achieves recognition accuracies of 92.52%, 92.38%, and 92.30%, as well as F1-scores of 91.92%, 92.13%, and 92.29% for the three fatigue states, affirming its reliability. This research provides technical support for the development of affordable and dependable wearable motion monitoring devices.

## 1 Introduction

Fatigue is a condition characterized by reduced work efficiency following extended or intense continuous work, often accompanied by subjective discomfort [1]. Prolonged periods of fatigue can lead to decreased work efficiency and a diminished quality of life [2]. Objective assessment of muscle performance using technological methods is crucial in mitigating muscle injuries. Surface electromyography (sEMG) detection and accelerometer (ACC) analysis technologies are rapidly advancing [3] due to their non-invasive, wearable, and cost-effective nature. The integration of these methods provides a more comprehensive kinematic estimation information [4].

While sEMG and ACC signals cannot directly analyze the extent of human fatigue, the bioelectrical signals within the human body undergo changes during fatigue. Researchers can determine the degree of fatigue by analyzing these signal variations. For instance, during fatigue, the mean power frequency (MPF) of sEMG signals decreases due to a slowdown in muscle fiber conduction velocity in “Relax”“Translation”“Tired” [5]. Simultaneously, the signal amplitude increases as more muscle fibers are recruited to maintain force output. Similarly, ACC signals during fatigue may exhibit increased head movement and delayed reaction times, reflecting the impact of fatigue on the nervous system’s response speed [6]. The fusion of sEMG and ACC signals is essential due to their complementary nature. This fusion helps overcome challenges associated with individual signals. For example, it mitigates issues like motion artifacts, electrode displacement, and noise interference in sEMG signals [7], and addresses concerns related to posture, gait, and tremors in ACC signals [8]. Ultimately, multi-feature fusion enhances the accuracy and robustness of fatigue detection, enabling its application in more complex scenarios [9].

Previous studies have explored various approaches for fatigue detection using sEMG and ACC signals. For instance, Ai et al. [10] proposed a method based on feature fusion and machine learning. They first extracted time-domain features and wavelet coefficients from sEMG signals and combined them with dynamic time features (e.g., mean, variance, zero-crossing rate) extracted from ACC signals. These fused features were then fed into classifiers such as Linear Discriminant Analysis (LDA) and Support Vector Machine (SVM) for fatigue state recognition. Their experimental results demonstrated that fusing sEMG and ACC signals could effectively improve the accuracy of fatigue recognition, achieving up to 90%. Zhang et al. [11] focused on lower limb motion pattern recognition and environmental feature estimation. They proposed a robust lower limb recognition system that utilized sEMG and ACC signals to identify different motion patterns (e.g., walking, running, going upstairs/downstairs) and estimate environmental features (e.g., ground slope, terrain type). Their research indicated that fusing sEMG and ACC signals could provide richer information, thereby enhancing the accuracy of motion pattern recognition and environmental feature estimation. Furthermore, Cheng et al. introduced a fall detection method based on an activity-aware framework [12]. This framework utilized sEMG and ACC signals to recognize users’ daily activities (e.g., standing, walking, sitting, falling) and detected fall events by analyzing changes in signal characteristics. Their study showed that fusing sEMG and ACC signals could effectively improve the sensitivity and specificity of fall detection, especially in daily activity monitoring environments. Fusing sEMG and ACC signals holds promising application prospects in various fields, including motion fatigue detection, motion pattern recognition, and fall detection.

Building upon early foundational research, this study combined sEMG and ECG signals [13]. By leveraging CNN, we achieved precise recognition of muscular fatigue during exercise, distinguishing between relaxed, transitional, and fatigued states with an accuracy exceeding 86%. Further research results indicated that the fatigue recognition model incorporating ECG outperformed single sEMG detection systems in terms of accuracy and stability. However, due to the complexity and cost of this system, it is not practical for regular sports training.

In an effort to reduce costs and develop fatigue detection devices tailored to general athletes, this paper introduces a multi-level attention mechanism that utilizes wearable sEMG and ACC signals to achieve precise sports fatigue recognition. The specific method involves the following steps: Firstly, classical time-domain and frequency-domain features are extracted from sEMG and ACC signals, which are then combined into a fused signal. Next, LFAM is introduced to enhance the waveform features of the fused signal. Subsequently, a fatigue classification model is established based on CNN with a DSAM. DSAM learns important information from two perspectives: channels and neurons, with minimal parameters introduced. Finally, the model performs type recognition for each sampling point through the fully connected layers.

The main contributions of this paper are threefold:

1. An innovative combination of multimodal signal fusion and a multi-level attention mechanism. This is achieved by fusing sEMG and ACC signals and introducing a multi-level attention mechanism within a CNN model.
2. The development of a Local Feature Attention Mechanism (LFAM) that effectively enhances the waveform characteristics of the signal. This, combined with the multi-level attention mechanism, allows for the capture of key features from multiple dimensions.
3. The establishment of a reliable method for recognizing fatigue states during exercise. This was achieved through rigorous testing and analysis involving healthy participants.

## 2 Materials and methods

### 2.1 Data acquisition

Prior to the study, all subjects will be informed of the study’s objectives and will have the option to withdraw participation at any time. Participants were provided with detailed information regarding the study’s content, purpose, methods, and potential risks, and they have volunteered to take part in the research. Written consent will be obtained from all participants before the study commences.

**Fig 1:**
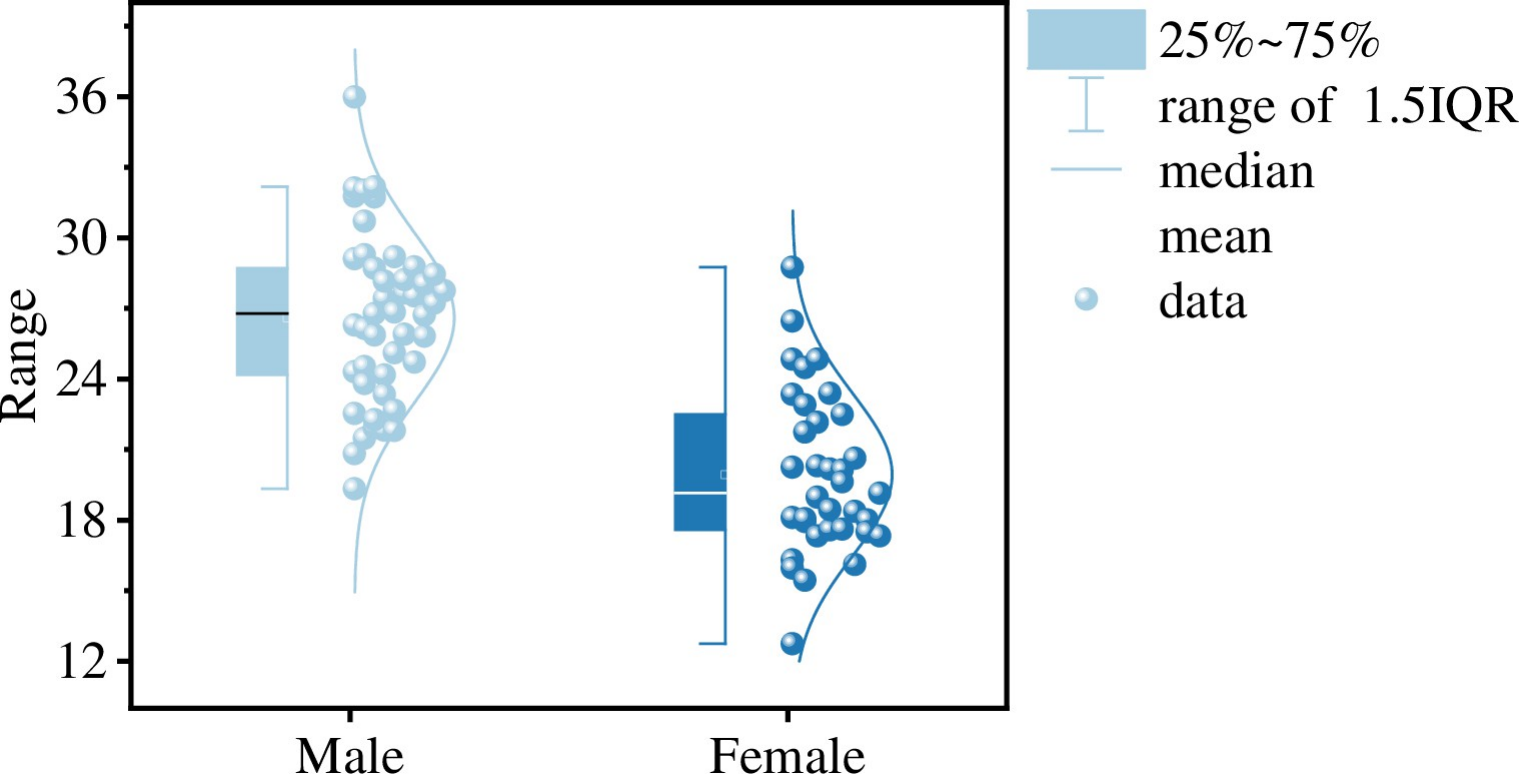
Distribution of participants’ BMI values.

This study recruited 80 healthy university students (45 males and 35 females) from East China University of Technology as participants, all of whom were right-handed. The recruitment criteria for participants included: normal or corrected-to-normal vision in both eyes, no history of drug abuse or psychiatric disorders, and no consumption of pain medications or psychiatric drugs in the past three months. Additionally, participants were required to have BMI values within the range shown in **Fig** 1. All participants were in good physical health with no history of cardiovascular diseases or severe muscle injuries. Furthermore, participants were instructed to refrain from consuming caffeine, nicotine, or alcohol, and to avoid engaging in vigorous exercise within 24 hours prior to the experiment.

To ensure precise regulation of exercise power, this study utilized the Lode Corival CPET (Lode B. V., Groningen, The Netherlands) cycle ergometer for exercise testing. sEMG were made of Ag-AgCl material and arranged in a bipolar configuration with a center-to-center distance of 20 millimeters and a diameter of 1 centimeter. The Noraxon Ultium® EMG sensor recorded sEMG signals at a sampling frequency of 2000 Hz, and data collection was conducted wirelessly using the Noraxon MR3 software. ACC signals were collected using a 9-axis IMU sensor (model WT901WIFIC) at a sampling rate of 20 Hz. Additionally, the Ultima GX system was used to measure participants’ ventilation (VE), oxygen consumption (VO_2_), and carbon dioxide production (VCO_2_) during the exercise tests. Prior to conducting the experiments, calibration of all sensors and experimental equipment was performed to ensure the accuracy of the data.

Following the recommendations for electrode placement on the gastrocnemius muscle (GA) outlined in the Surface Electromyography for the Non-Invasive Assessment of Muscles (SENIAM) guidelines [14], sEMG sensors were securely attached using elastic straps. Prior to testing, participants were familiarized with the experimental equipment and procedures. Each participant completed a 3-minute warm-up on a cycle ergometer, followed by an incremental exercise test conducted in a temperature-controlled environment (26 ± 3°C). The initial workload was set at 100 W, with increments of 25 W applied every minute. Participants were instructed to maintain a cycling cadence between 70–75 revolutions per minute (rpm) throughout the test. The test was terminated when a participant was unable to maintain a cadence of 70 rpm or exhibited clear signs of fatigue. Each participant completed the same testing protocol on three consecutive days with a minimum 24-hour rest interval between sessions to ensure adequate recovery. The anaerobic threshold (AT) was determined using the V-slope method from the oxygen consumption (VO2) and carbon dioxide production (VCO2) data collected during the test. The sEMG and ACC data were synchronized with the AT and categorized into three phases: Easy (start of test to AT), Transition (AT to 50% of the time between AT and test termination), and Tired (50% of the time between AT and test termination to test termination) [15].

### 2.2 Signal preprocessing

Signal preprocessing is crucial to enhance the accuracy of the classification model. Raw sEMG and ACC signals typically contain various types of noise, such as physiological noise, power noise (50 Hz), and baseline noise (e.g., thermal noise, electrochemical noise, and motion artifacts). The preprocessing of sEMG signals involves differential amplification, high-pass filtering, low-pass filtering, signal amplification, and signal tracking. In this process, cascaded high-pass and low-pass filtering is utilized to preserve the frequency band ranging from 20 Hz to 500 Hz. Additionally, signal amplification and tracking steps are employed at the conclusion of preprocessing to further amplify the small raw signals, enhancing sampling accuracy. Subsequently, the collected signals are segmented into multiple epochs, each lasting for 10 seconds. These epochs are utilized to assess the participant’s movement status. These preprocessing steps are essential for noise elimination and to prepare the signals for subsequent classification and analysis.

### 2.3 Feature-level data fusion

The objective of feature-level data fusion is to reduce the dimensionality of feature vectors while preserving essential information. In this study, after signal processing, the sEMG data and ACC data are fused at the feature level. For sEMG signals, typical time-domain and frequency-domain features are selected, as listed in **Table** 1. These features have been chosen based on previous research and effectively capture various properties of sEMG and ACC signals. They are commonly utilized in areas such as fatigue assessment, gesture recognition, and motion intent recognition. Given that both sEMG and ACC devices have multiple channels, the widely used 6-channel and 18-channel signals are selected for this study. Consequently, the final sEMG feature map comprises 6×42 features, while the ACC feature map comprises 18×6 features. By transposing the ACC feature map and concatenating it with the sEMG feature map, a feature map of 6×60 is obtained. This feature-level fusion strategy integrates information from both signals and provides an increased number of input features for the multi-level attention mechanism.

**Table 1:** Typical feature list of sEMG and ACC signals.

sEMG Features	Name of Variables	Reference	Channels× Dimensions	ACC Features	Name of Variables	Reference	Channels× Dimensions
mDWT	marginal of discrete wavelet transform	Lucas *et al*. [16]	6×8	MEAN	mean	Nichols *et al*. [17]	18×1
HIST	histogram of sEMG	Ayachi *et al*. [18]	6×20	VAR	variance	Nichols *et al*. [17]	18×1
RMS	root mean square	Abdelouahad *et al*. [19]	6×1	RMS	root mean square	Nichols *et al*. [17]	18×1
ZC	zero crossing	Conradsen *et al*. [20]	6×1	WL	waveform length	Khan and Kwon [21]	18×1
SSC	slope sign change	Sayin *et al*. [22]	6×1	MAV	mean absolute	Alberts *et al*. [23]	18×1
WL	waveform length	Sayin *et al*. [22]	6×1	MAVS	MAV slope	Alberts *et al*. [23]	18×1
MAV	mean absolute value	Abdelouahad *et al*. [19]	6×1				
MAVS	MAV slope	Ryu *et al*. [11]	6×1				
WAMP	Willison amplitude	Ryu *et al*. [11]	6×1				
ARCs	autoregressive coefficients	Emayavaramban *et al*. [24]	6×1				
MNF	mean frequency	Corvini *et al*. [25]	6×1				
PSR	power spectrum ratio	Maisetti *et al*. [26]	6×1				
Total			6×42	Total			18×6

### 2.4 Multi-level attention mechanism fusion

This study tackles the multidimensional data resulting from feature fusion by implementing a multi-level attention mechanism. It assigns scores and weights to the input sequences, network channels, and neurons individually, thereby amplifying the impact of crucial features from different dimensions on the model’s outcomes.

#### 2.4.1 Local feature attention mechanism

To enhance the local features of the signals, this paper utilizes an attention mechanism based on the envelope of the amplitude. This mechanism is employed for input preprocessing in the model. It automatically adjusts attention weights based on the amplitude variations in the input sequences, thus enhancing local waveform features. The envelope of the amplitude of the input signal, denoted as d(t), is obtained through the Hilbert transform, as shown in Eq (1):

$$H[d(t)]=\frac{1}{\pi }\int_{-\infty }^{+\infty }\frac{d(\tau )}{t-\tau }d\tau$$ (1)

Where the Hilbert transform result *d*(*t*) can be used to construct an analytic function *d*_*a*_(*t*), as shown in Eq(2)

$$d_{a}(t)=d(t)+iH[d(t)]=A(t)e^{i\theta (t)}$$ (2)

Where *A*(*t*) and *θ*(*t*) is the instantaneous amplitude and instantaneous phase angle of *d*(*t*), *A*(*t*) is the envelope of the amplitude, calculated as shown in Eq(3)

$$A(t)=\sqrt{{\left[d(t)\right]}^{2}+{\left[H[d(t)]\right]}^{2}}$$ (3)

**Fig 2:**
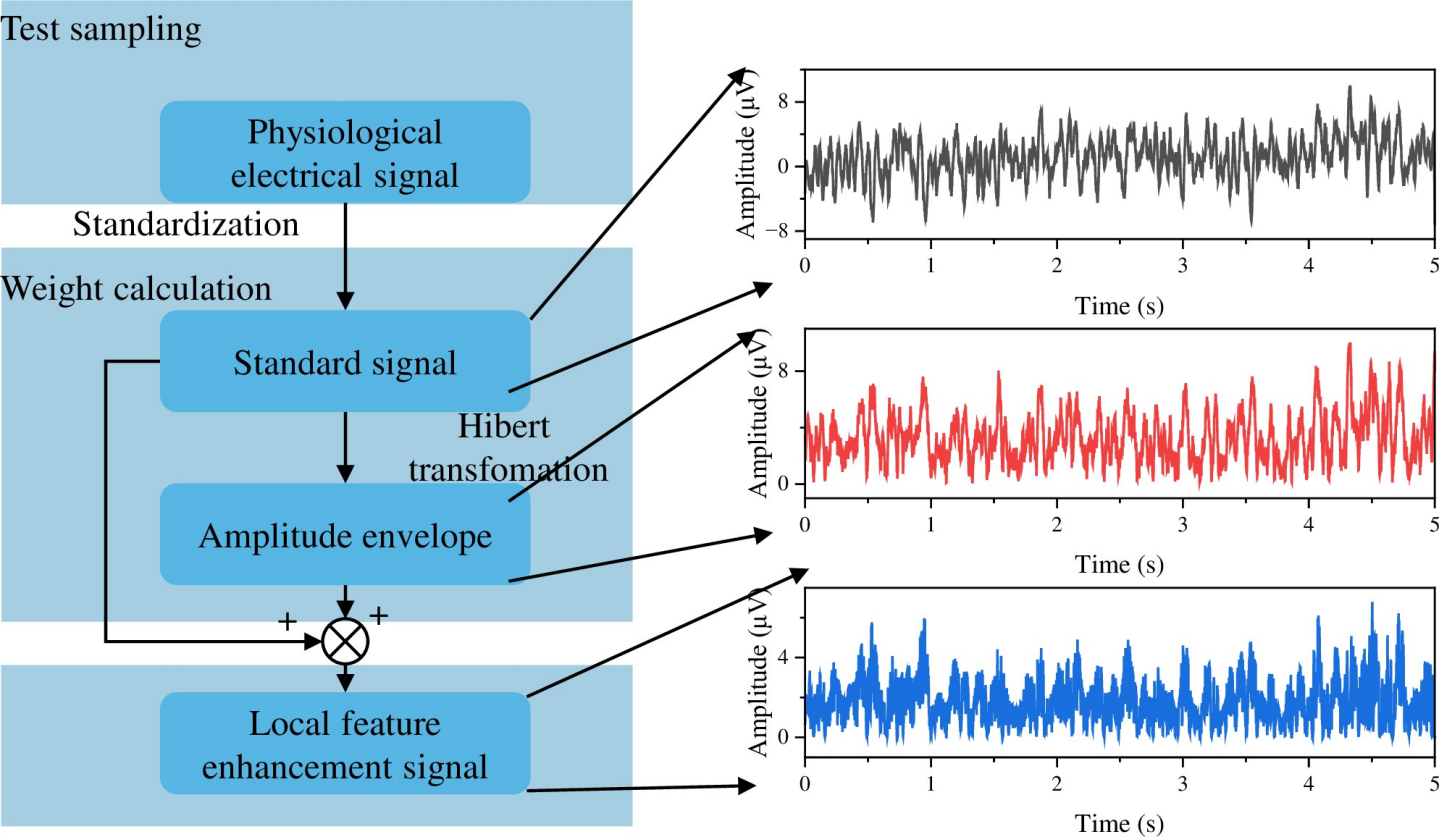
Flowchart of LFAM for feature fusion.

This method offers the advantage of accurately reflecting the local perturbation characteristics of bioelectric signals and implementing an attention mechanism for local waveform features. The mechanism consists of two main steps: weight calculation and weighted computation, with the process depicted in **Fig** 2. Firstly, the input information undergoes standardization and is then transformed into the amplitude envelope through the Hilbert transform. Finally, the envelope sequence is used as attention weights for weighted computation along with the standardized signal. For physiological signals, in a relaxed state, the envelope remains relatively stable, and the corresponding weight values are approximately constant at 1. Therefore, the weighted signal remains largely unchanged. As the exercise progresses, the signal’s amplitude fluctuates, and the envelope exhibits variations. Consequently, the weight values enhance the local features of the waveform.

#### 2.4.2 Dual-scale attention mechanism

**Fig 3:**
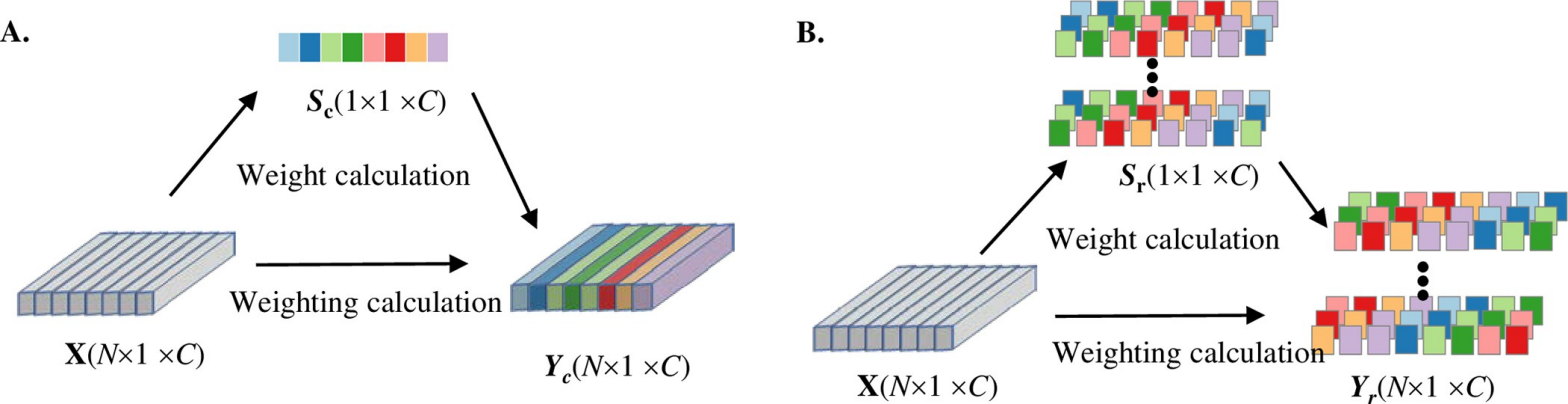
DSAM flowchart. A. Channel attention mechanism. B. Neuron attention mechanism.

The attention mechanism scores elements based on their importance to focus on critical information. For the multi-channel data **X** after convolutional computation, the respective channel and neuron attention mechanisms are illustrated in Fig 3. Single-scale attention mechanisms struggle to consider the importance of features across multiple dimensions. Therefore, this paper proposes a DSAM based on channels and neurons. The channel attention mechanism enhances the correlation between different channels, while the neuron attention mechanism enhances the representational capacity of each neuron in the feature map.

The channel attention mechanism is implemented by SENet and primarily assesses the importance of channels through compression and activation operations. Firstly, it utilizes Global Average Pooling (GAP) to compress each channel, resulting in the feature vector **x**_*sq*_, as calculated in Eq(4):

$$x_{sq{\_}^{c}}=F_{sq}(X_{c})=\frac{1}{N}\sum_{i=1}^{N}X_{c}(n)$$ (4)

Where *N* represents the length of single-channel data, ***X***_*c*_ and x_sq_c_ are the data and compressed feature values for channel *c*. Then, for the feature vector *F*_*ex*_, an activation operation is performed, which involves scaling through two fully connected layers. The resulting weight coefficient *s* is used to assess the importance of each channel feature. The specific calculation process is as shown in Eq (5):

$$\begin{array}{c} s=F_{ex}(X_{sq},W)\\ =sigmoid(g(X_{sq},W))\\ =sigmoid(W_{2}\cdot \mathrm{ReLU}(W_{1}X_{sq}))\\ W_{1}\in R^{\frac{C}{r}\times C},\;W_{2}\in R^{C\times \frac{C}{r}} \end{array}$$ (5)

Where **W**_1_ and **W**_2_ are scaling layer coefficient matrices and *r* is the scaling factor. The channel weight coefficient *s* is multiplied by the corresponding channel data, completing the attention-weighted calculation

To capture high-dimensional detailed features of the signals, this paper introduces the neuron attention mechanism of simAM. It finds key nodes by measuring the linear separability of neurons without adding additional parameters. Neuron energy is a critical parameter in this mechanism for distinguishing the importance of different neurons. The calculation is as follows, as shown in Eq (6), (7), and (8):

$$e_{q}(w_{q},b_{q},y,x_{i})={\left(y_{q}-\hat{q}\right)}^{2}+\frac{1}{N-1}\sum_{i=1}^{N-1}{\left(y_{o}-{\hat{x}}_{i}\right)}^{2}$$ (6)

$$\hat{q}=w_{q}q+b_{q}$$ (7)

$${\hat{x}}_{i}=w_{q}x_{i}+b_{q}$$ (8)

Where *q* and *x*_*i*_ represent the target neuron and other neurons in the same channel, with *i* as the index of neurons in the channel. The lower the energy of the target neuron, the better it reflects the linear separability of neuron *q* from other neurons, so the goal is to minimize the above equation. To facilitate optimization, binary labeling is applied to *y*_*q*_ and *y*_*o*_, and a regularization term is added to the objective function, transforming the expression into Eq (9):

$$e_{q}(w_{q},b_{q},y,x_{i})=\frac{1}{N-1}\sum_{i=1}^{N-1}(-1-(w_{q}x_{i}+b_{q}){)}^{2}+(1-(w_{q}q+b_{q}))+\lambda {w_{q}}^{2}$$ (9)

To alleviate the computational burden of calculating different neurons *e*_*q*_ in different channels, *w*_*q*_ and *b*_*q*_ are obtained through an analytical solution, as shown in Eq (10):

$$w_{q}=-\frac{2(q-\mu_{q})}{{\left(q-\mu_{q}\right)}^{2}+2\sigma_{q}^{2}+2\lambda }$$ (10)

$$b_{q}=-\frac{(q+\mu_{q})w_{q}}{2}$$ (11)

Where *μ*_*q*_ and *σ*_*q*_ represent the mean and variance of all neurons in a single channel except the target neuron *q*. Since neurons within the same channel have a consistent distribution, the calculation of mean and variance for the target neuron *q* can be simplified by calculating statistical parameters based on all neurons within the channel as a whole.


$$\{\begin{array}{c} \hat{\mu }=\frac{1}{N}\sum_{i=1}^{N}x_{i}\\ {\hat{\sigma }}^{2}=\frac{1}{N}\sum_{i=1}^{N}{\left(x_{i}-\hat{\mu }\right)}^{2} \end{array}$$ (12)

The final minimum energy function for the target neuron *q* can be calculated using Eq (13):

$$e_{q}^{*}=\frac{4({\hat{\sigma }}^{2}+\lambda )}{{\left(q-\hat{\mu }\right)}^{2}+2{\hat{\sigma }}^{2}+2\lambda }$$ (13)

The smaller the minimum energy function value of the neuron, the more prominent the features it contains. Therefore, 1/e*_q_ represents the importance of each neuron. The weighted calculation of this attention mechanism is performed based on the importance of each neuron as shown in Eq(14):

$$Y_{r}=sigmoid(\frac{1}{E})⊙X$$ (14)

Where **E** represents the matrix of minimum energy function values for all neurons. The *sigmoid* activation function is used to prevent excessively large values in the parameter matrix. The pseudo-code for Multi-level Attention Mechanism Fusion is as follows S1 Algorithm. The pseudo-code for DSAM is as follows S2 Algorithm.

### 2.5 ACC and sEMG fatigue prediction classification model

**Fig 4:**
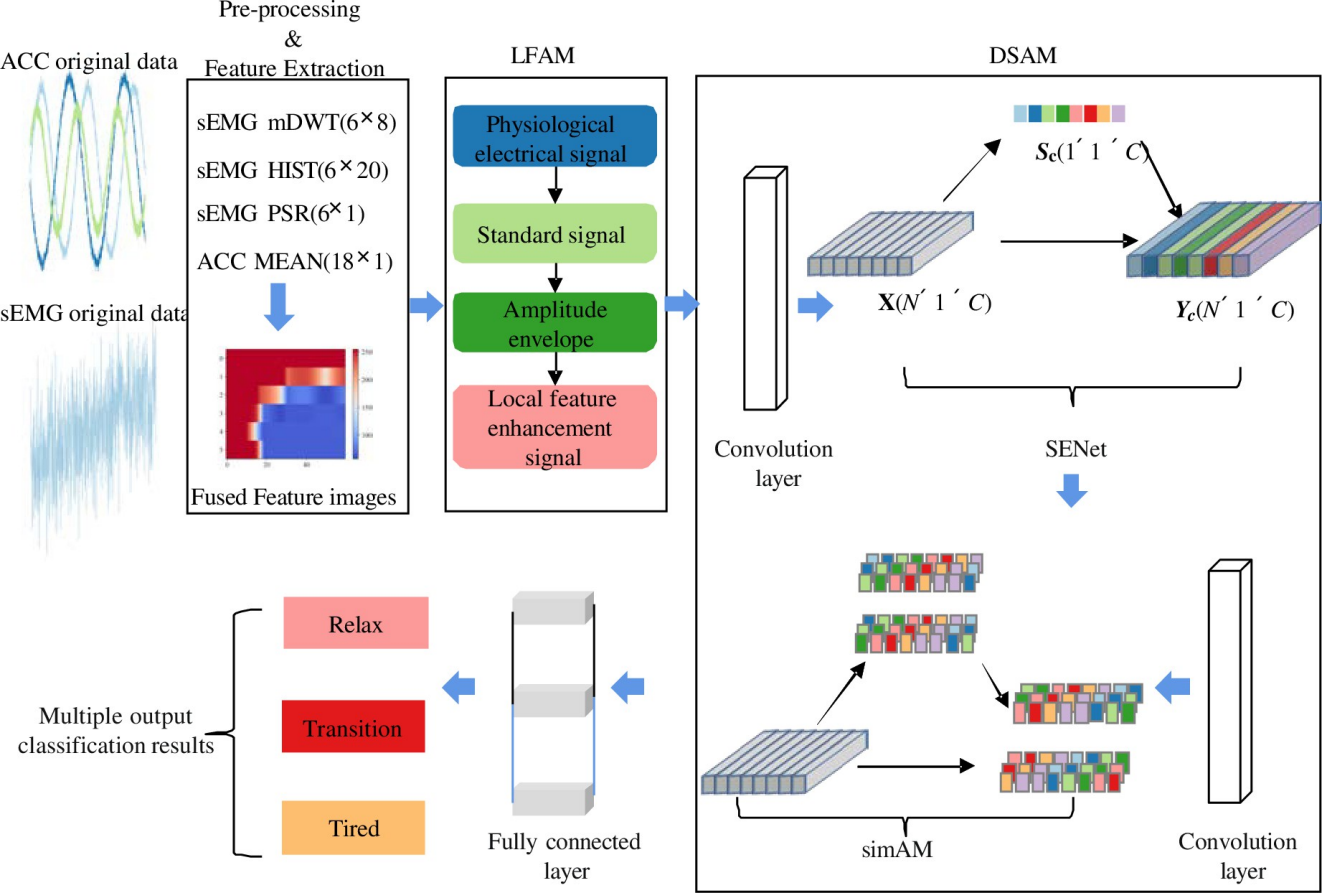
Structure diagram of sEMG and ACC fatigue prediction classification model.

The framework of the sEMG and ACC fatigue prediction classification model is illustrated in Fig 4. It comprises three key components: data preprocessing, multi-level attention mechanism, and the classification model. In the data preprocessing phase, a LFAM is utilized to enhance the waveform characteristics of the signals, thereby improving the efficiency of feature extraction related to the motion state for the subsequent classification model. In the multi-level attention mechanism, channel and neuron attention mechanisms are hierarchically integrated, taking into account the distribution patterns of feature values in the CNN layers. In the shallow convolutional layers, where the feature space distribution is less distinct, the channel attention mechanism is applied for broader focus, reducing redundancy and enhancing the classification model’s efficiency. Conversely, in the deep convolutional layers containing more semantic information, the neuron attention mechanism is used to extract the most distinctive features, effectively enhancing the model’s learning capacity. Considering the significance of temporal characteristics in the data, pooling layers in the CNN have been omitted. Regarding the integration of attention mechanisms, after several tests, it was determined that configuring the neuron attention mechanism in the final convolutional layer while employing channel attention mechanisms in the other layers achieved the optimal balance between efficiency and accuracy.

The tunable parameters of this model primarily focus on the DSAM and the convolutional layers. We need to adjust the scaling factor (*r*) for SENet and the regularization coefficient (*λ*) for simAM. After multiple rounds of testing, we have set *r* to 18 and *λ* to 0.0001 as the optimal values. As for the convolutional layers, their parameters need to be determined based on the characteristics of the signals and the model’s task. Considering the existence of both long-term steady-state and transient disturbance features in ACC and sEMG signals, reflecting changes in motion states, we must configure convolutional kernels of various sizes to adapt to the uncertain proportion of disturbance features in the input sequences. Additionally, due to individual differences in sEMG and ACC signals, we need to increase the depth of the convolutional layers to ensure classification accuracy. The convolutional layers in this study are divided into two groups: one group includes large-sized convolutional kernels, and the other group includes small-sized convolutional kernels. The final parameter configuration has been determined through iterative testing, and specific numerical values can be found in **Table** 2.

**Table 2:** Model parameter settings.

Layer name	Output size	Parameter settings	Activation function	Layer name	Output size	Parameter settings	Activation function
LFAM	6×60	None	None	Conv5	6×60	*f* = 100, *k* = 2, *s* = 1	ReLU
Conv1	6×60	*f* = 100, *k* = 2, *s* = 1	ReLU	SENet5	6×60	*r* = 16	ReLU+Sigmoid
SENet1	6×60	*r* = 16	ReLU+Sigmoid	Conv6	6×60	*f* = 100, *k* = 2, *s* = 1	ReLU
Conv2	6×60	*f* = 100, *k* = 2, *s* = 1	ReLU	SENet6	6×60	*r* = 16	ReLU+Sigmoid
SENet2	6×60	*r* = 16	ReLU+Sigmoid	Conv7	6×60	*f* = 100, *k* = 2, *s* = 1	ReLU
Conv3	6×60	*f* = 100, *k* = 2, *s* = 1	ReLU	SENet7	6×60	*r* = 16	ReLU+Sigmoid
SENet3	6×60	*r* = 16	ReLU+Sigmoid	Conv8	6×60	*f* = 100, *k* = 2, *s* = 1	ReLU
Conv4	6×60	*f* = 100, *k* = 2, *s* = 1	ReLU	simAM	6×60	*λ* = 0.0001	Sigmoid
SENet4	6×60	*r* = 16	ReLU+Sigmoid	Dense	6×60	units = 16	Softmax

*f is the number of convolutional kernels, *k* is the size of the convolutional kernel, *s* is the convolutional kernel’s stride.

### 2.6 Model evaluation parameters

The model’s training was performed using 10-fold cross-validation, which involves randomly dividing the dataset into 10 parts, using 9 parts for training, and 1 part for validation in each iteration. This process was repeated 10 times, each time with a different training set and validation set. Finally, the results from these 10 iterations were averaged to estimate the algorithm’s accuracy. This method provides a comprehensive assessment of the model’s performance. In the results analysis, various evaluation metrics were employed, including *Accuracy*, *Precision*, *Recall*, and *F1-Score*, as the primary indicators. These metrics help evaluate the model’s performance in classification tasks and provide a comprehensive assessment of its performance. Accuracy represents the proportion of correctly identified motion states in ACC and sEMG signals and focuses on the correctness of all prediction results. It is calculated as shown in Eq (15):

$$Accuracy=\frac{TP+TN}{TP+TN+FP+FN}\times 100\%$$ (15)

Precision refers to the ratio of the number of samples correctly classified as positive (e.g., easy, transition, fatigue) to the total number of samples classified as positive by the model. Precision for each category can be calculated using Eq (16):

$$Precison=\frac{TP}{TP+FP}\times 100\%$$ (16)

Recall, also known as sensitivity or true positive rate, is the ratio of the number of samples correctly classified as positive (e.g., easy, transition, fatigue) to the total number of actual positive samples. Recall for each category can be calculated using the following Eq (17):

$$Recall=\frac{TP}{TP+FN}\times 100\%$$ (17)

F1-score is a metric that combines precision and recall into a single value, taking their harmonic mean. It is calculated using Eq (18):

$$F1‐score=\frac{2*Precision*Recall}{Precision+Recall}$$ (18)

The explanations of each metric are as follows: *TP* (True Positives): The number of samples correctly classified as positive, meaning the actual positives that were correctly predicted as positives by the model. *TN* (True Negatives): The number of samples correctly classified as negative, meaning the actual negatives that were correctly predicted as negatives by the model. *FP* (False Positives): The number of samples incorrectly classified as positive, meaning the actual negatives but were erroneously predicted as positives by the model. *FN* (False Negatives): The number of samples incorrectly classified as negative, meaning the actual positives but were erroneously predicted as negatives by the model. These metrics are used to assess the performance of a classification model, helping to measure the model’s ability to recognize positives and negatives, as well as performance metrics such as accuracy, precision, and recall.

All models involved in this paper are constructed using the Keras framework. The specific software and hardware configurations are shown in Table 3.

**Table 3:** Hardware models and software versions.

Hardware	Models	Software	Version
CPU	Intel Core i5-13500H	Python	3.11.4
GPU	NVIDIA GeForce RTX	Keras	2.13.1
RAM	DDR5-6400	TensorFlow	2.13.0
HDD	1 TB PCIe 4.0	CUDA	10.0

## 3 Experiments and results

### 3.1 Model training and ablation experiments

**Fig 5:**
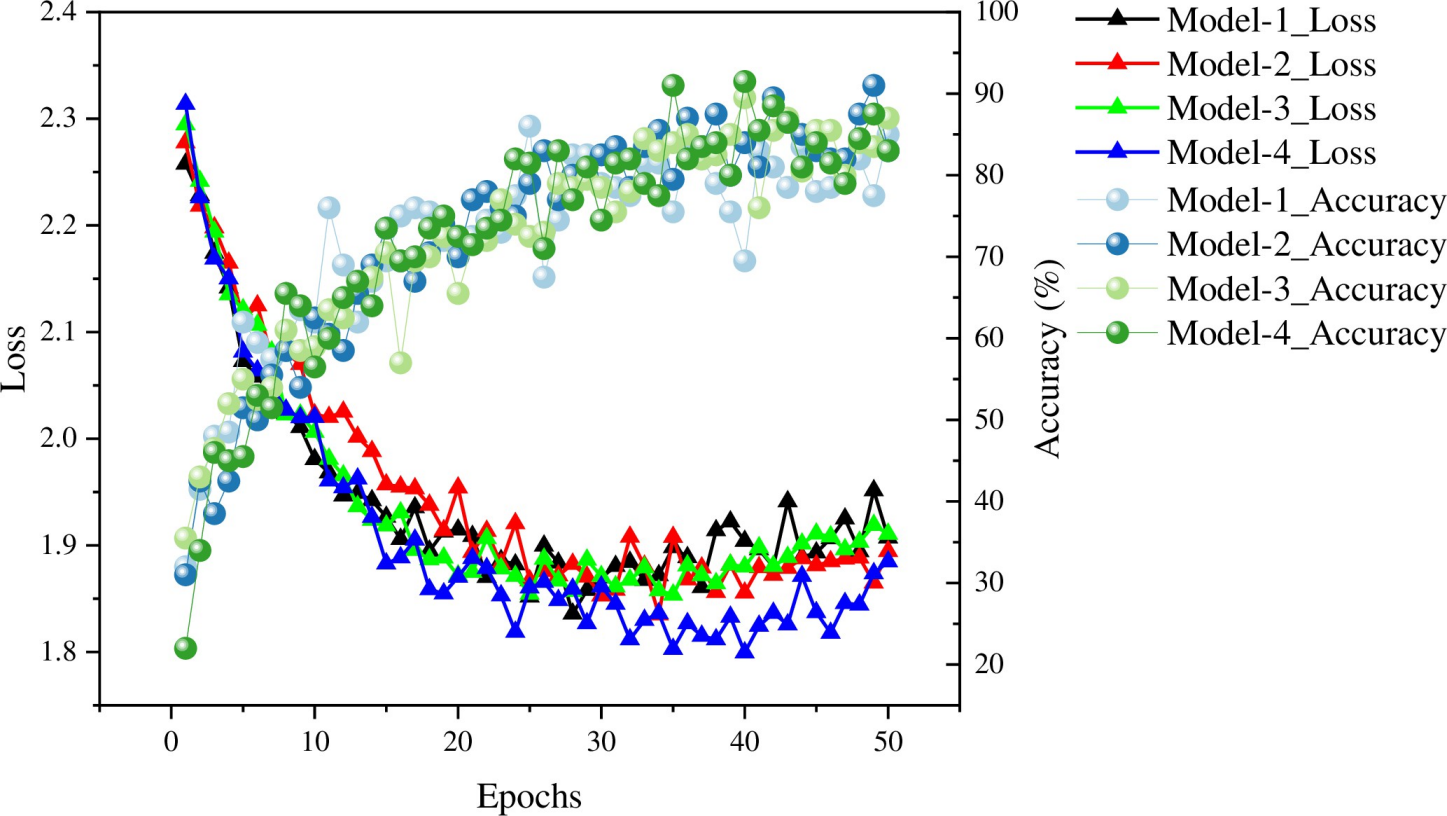
The training process of each classification model.

This section conducts ablation experiments to demonstrate the training process of the model, which aids in evaluating the model’s training performance and emphasizing the significance of various modules. The experiments utilized a controlled variable approach to establish four comparative models: Model-1 (CNN optimized with LFAM), Model-2 (CNN optimized with SENet), Model-3 (CNN optimized with simAM), and Model-4 (CNN without any attention mechanism). These models maintained consistent hyperparameter settings. Following training, a comparative analysis of their loss values and accuracy was conducted. Moreover, attention was focused on the changes in the training and validation sets throughout the training process, as illustrated in **Fig** 5. The design of ablation experiments facilitates a comprehensive understanding of the roles of different optimization modules in the model and their contributions to performance enhancement. By comparing the training outcomes of different models, valuable insights can be gained regarding the most critical optimization strategies for enhancing model performance. This analysis guides further enhancements and optimizations of the model to achieve superior performance levels.

In order to further understand the statistical relationship behind the data, the variance test is done for the results of each model, The results are presented in S1 Table. The CNN model (Model 4) that did not pay attention to the mechanism optimization in the training process performed poorly in terms of accuracy and convergence speed. After optimization with LFAM (Model-1), the classification accuracy and convergence speed of the model are improved, but the model experiences misfitting, that is, the accuracy on the validation set is lower than that on the training set. For models optimized with SENet (Model-2) and DSAM (Model-3), their accuracy is improved by about 5.2% and convergence is faster. However, the accuracy analysis of the training set and the validation set shows that both models have the problem of overfitting. In conclusion, the optimization of LFAM, channel attention mechanism and neuron attention mechanism significantly improve the performance and convergence speed of CNN model. Among them, the model optimized by channel mechanism and neuron attention mechanism has a low degree of overfitting. Ablation experiment results show that the proposed optimization strategy effectively enhances the generalization ability of ACC and surface EMG fusion classification model.

S1 and S2 Figs show the mean changes of Accuracy and Loss under different models. At < 0.05 level, the differences in Accuracy and Loss between models were small and not significant. The advantage of Model-1 is that it has the lowest average loss and the lowest standard deviation. The advantage of Model-2 is that it has the highest accuracy and a higher recall rate. The disadvantage of Model-3 is that it has a higher standard deviation and larger individual differences. Through variance analysis, it was determined that Model-1 and Model-2 may be more suitable for the analysis of sEMG and ACC.

### 3.2 Pseudo-artifact robustness testing

**Fig 6:**
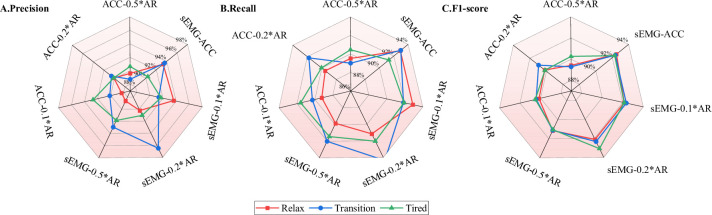
Model recognition performance in different pseudo-artifact environments. A. Accuracy, B. Precision, C. Recall, D. F1-score.

Because ACC and sEMG signals mutually contain artifacts, robustness testing against artifacts is a critical step in practical applications. Due to significant individual differences and varying weights of sEMG artifacts in ACC signals, different weights were assigned to form classification models. Comparing and analyzing the differences between models can assess the generalization ability of the proposed method. ACC data from the X, Y, and Z axes were assigned weights of 0.1, 0.2, and 0.5, respectively, to enhance the sEMG data, creating artifact combinations like sEMG-0.1*AR, sEMG-0.2*AR, and sEMG-0.5*AR. Conversely, sEMG signals were assigned weights of 0.1, 0.2, and 0.5 to ACC data from the X, Y, and Z axes, respectively, creating models like ACC-0.1*AR, ACC-0.2*AR, ACC-0.5*AR, and the artifact-free sEMG-ACC. The CNN models optimized by the LFAM, channel attention mechanism, and neuron attention mechanism from the previous section were applied to these four models. To objectively assess the multi-class performance of each model, Accuracy, Precision, Recall, and F1-score were used to evaluate their generalization ability. These four metrics are widely used in multi-class problems: accuracy measures the correctness of all predictions, precision focuses on the accuracy of positive predictions, recall emphasizes the proportion of true positive samples predicted correctly, and the F1 score reflects the balance between precision and recall. Due to the large amount of data, these metrics will be presented as weighted averages. The test results for all models are shown in the Fig 6 and S2 **Table**. Higher values for each metric indicate better classification performance of the model.

The sEMG-0.1*AR model exhibits high accuracy, precision, and F1-score in the Relax state but performs poorly in the Tired state, the confusion matrix is shown in S3 Fig. The sEMG-0.2*AR model performs best in the Tired state with an accuracy of 93.09%, precision of 90.76%, and F1-score of 90.25%, but its performance decreases in other states. The sEMG-0.5*AR model shows relatively stable performance across different states but overall performs slightly worse than the sEMG-0.2*AR model. The ACC-0.1*AR model remains relatively stable, especially excelling in the Translation state, but its performance is slightly lower in the Tired state. The ACC-0.2*AR model performs best in the Relax state but fluctuates in performance in other states. The ACC-0.5*AR model maintains stable performance with the highest accuracy of 90.28%, precision of 90.48%, and F1-score of 91.01% in the Relax state. The sEMG-ACC model exhibits overall stability, with accuracy ranging from 92.30% to 92.52% in Relax, Transition, and Tired states, and the highest F1-scores in these states. These results highlight the significant impact of different motion states on classification model performance. The choice of model should be tailored to specific requirements. These findings provide valuable insights into the performance of classification models under different motion states, offering guidance for motion classification tasks.

### 3.3 Computational analysis

The computational analysis is a crucial task for the application of new methods to practical wearable devices. This section records the training and testing times from sections 3.1 and 3.2 separately, presenting them in the form of averages in Table 4.

**Table 4:** Computational analysis of each model.

Models	Total epochs	Average training time/ epoch (s)	Average testing time / sample (ms)
Model-1	46	31.72	5.95
Model-2	36	36.18	6.54
Model-3	38	38.25	8.81
Model-4	68	30.22	12.82
sEMG-0.1*AR	37	37.11	6.58
sEMG-0.2*AR	38	37.45	6.98
sEMG-0.5*AR	38	37.87	6.88
ACC-0.1*AR	37	37.54	6.48
ACC-0.2*AR	37	37.45	6.87
ACC-0.5*AR	38	37.98	6.89
sEMG-ACC	36	37.74	6.84

From the results, the following conclusions can be drawn:

①Model-4 outperforms other convolution models in terms of training and testing time, despite the absence of attention mechanisms, resulting in a larger overall algebraic size.

②Among Model-1, Model-2, and Model-3, LFAM optimization in Model-1 leads to the shortest training and testing times as it does not introduce additional parameters or entail more loop operations.

③In the case of simAM, although no additional parameters are introduced, it involves more computational operations, leading to longer training and testing times compared to Model-1.

④Model-3, incorporating simAM, exhibits longer training and testing times than SENet.

⑤Comparison of models integrating three attention mechanisms and artifacts reveals that the training and testing times primarily fall between Model-2 and Model-3. The inclusion of artifact weights has minimal impact on training and testing times and does not significantly alter the overall algebraic size.

⑥The computational requirements of the models appear suitable for practical applications based on the above findings.

### 3.4 Compare with other classification methods

This section selects commonly used methods for sEMG and ACC fatigue state recognition, including Feature Fusion Network (FFNET). This method combines one-dimensional feature extraction techniques of sEMG and ACC, such as Root Mean Square (RMS), Average Amplitude Change (AAC), and Mean Absolute Value (MAV), with two-dimensional extraction techniques (such as Fast Fourier Transform). It has certain advantages [22]. Another method is the multimodal fusion strategy, Hybrid Fusion (HyFusion), [23]which is a fusion framework and a novel and practical multi-scale model. It consists of parallel intra-modality and inter-modality branches. Both methods are implemented in Python and demonstrated to be effective on the original public dataset. In addition, the commonly used kernel regularized least squares (KRLSs) [19], support vector machine SVM [24], and the state-of-the-art word (MV-CNN) [25] were established.

The Table 5 and S4 Fig provided compares the performance of five different modeling methods for classifying three states. FFNET performs similarly to Model this paper in terms of accuracy and F1 score, but slightly worse in terms of precision and recall. The performance of HyFusion, MV-CNN, KRLSs, and SVM is relatively weak, especially in terms of accuracy and F1 score. The advantage of FFNET lies in its ability to extract features from sEMG and ACC signals. These features have been verified by prior experience and are relatively effective. Feature fusion can improve the performance of the model, but this method is sensitive to noise data. In contrast, the method proposed in this paper first performs denoising on the data, then extracts features, and then establishes a multi-level fusion model. The advantages are obvious.

**Table 5:** Comparison of statistical parameters of different methods.

Model	State	Accuracy	Recall	Precision	F1-score
FFNET	Relax	0.904	0.912	0.934	0.923
FFNET	Transition	0.901	0.903	0.934	0.918
FFNET	Tired	**0.908**	**0.925**	0.875	0.899
HyFusion	Relax	0.901	0.903	0.914	0.908
HyFusion	Transition	0.900	0.901	0.895	0.898
HyFusion	Tired	0.900	0.899	0.894	0.897
KRLSs	Relax	0.878	0.834	0.751	0.790
KRLSs	Transition	0.867	0.802	0.869	0.834
KRLSs	Tired	0.892	0.875	0.905	0.890
SVM	Relax	0.878	0.834	0.757	0.794
SVM	Transition	0.871	0.812	0.847	0.829
SVM	Tired	0.886	0.859	0.914	0.886
MV-CNN	Relax	0.901	0.902	0.780	0.837
MV-CNN	Transition	0.892	0.875	0.899	0.887
MV-CNN	Tired	0.866	0.799	0.918	0.855
Our method	Relax	**0.908**	0.924	**0.935**	**0.929**
Our method	Transition	**0.908**	0.923	0.926	0.925
Our method	Tired	**0.908**	0.924	0.910	0.917

## 4 Conclusion

To develop a cost-effective and dependable sports fatigue detection device, this study introduces a novel approach by fusing sEMG and ACC features to collectively analyze the fatigue levels of subjects. Furthermore, it proposes a classification methodology leveraging multi-level attention mechanisms, incorporating both LFAM and DSAM to improve the processing of high-dimensional data comprising sEMG and ACC features. The conclusions derived from model training, robustness analysis, artifact robustness testing, and computational analysis are as follows:

Upon comparing the waveform features before and after LFAM processing, it is evident that LFAM enhances the waveform features, underscoring its utility in the preprocessing stage for subsequent feature extraction and learning. This observation is consistent with prior research on LFAM, as documented in [27]. Regarding computational analysis, the incorporation of LFAM does not significantly prolong training and testing times, suggesting minimal additional internal operations introduced to the model. The integration of DSAM enables the model to learn feature importance from both channel and neuron perspectives, integrated into the CNN model in a hierarchical fusion approach. Through ablation experiments and artifact inclusion trials, this optimized model showcases superior recognition accuracy and training efficiency. Remarkably, even with the incorporation of artifacts in the fusion data of ACC and sEMG, the model maintains strong generalization capabilities, demonstrating robust performance.

Based on the thorough comparative analyses presented in this paper, the proposed multi-level attention mechanism fusion classification model adeptly integrates ACC and sEMG features to classify multi-dimensional data. Through rigorous ablation experiments, artifact inclusion studies, and computational analyses, it has been evident that the implementation of this model does not substantially escalate computational burden. This enhanced robustness equips the model to effectively address the interferences stemming from sEMG and ACC artifacts. Consequently, this model holds the potential to aid in discerning individual fatigue variations based on ACC and sEMG signals.

Drawing on previous research involving sEMG and ECG, alongside the current investigation into sEMG and ACC signals, our team is committed to addressing the challenges of fatigue monitoring during physical activities utilizing portable wearable devices. We have explored a range of traditional methods and their combinations to enhance recognition accuracy. The fusion of ACC and sEMG signals in this endeavor has yielded promising outcomes, with the incorporation of multi-level attention mechanisms further bolstering classification performance. Looking ahead, our research group aims to implement this algorithm into embedded devices, creating compact wearable devices for monitoring factors like fatigue, injury, stroke, and beyond on athletes’ legs.

## Supporting information

S1 Table Tukey’ HSD of Loss value during training of different classification models. (DOCX)

S2 Table Bi-factor variance accuracy analysis of different methods and exercise states. (DOCX)

S1 Fig Average loss of different classification models. (PDF)

S2 Fig Box plot of accuracy for different classification models. (PDF)

S3 Fig Model confusion matrix diagram in different pseudo-artifact environments. A.sEMG-ACC, B.sEMG-0.1*AR, C.sEMG-0.2*AR, D.sEMG-0.5*AR, E.ACSI-0.1 *AR, F.ACSI-0.2 *AR, G.ACSI-0.5 *AR.
(PDF)

S4 Fig The confusion matrix of the model is established by different methods. A. FFNET, B. HyFusion, D. KRLSs, E.MV-CNN, F.Our method.
(PDF)

S1 Algorithm Multi-level Attention Mechanism Fusion (MAMF). (DOCX)

S2 Algorithm Dual-scale Attention Mechanism (DSAM). (DOCX)
