# Supplementary material for: Multilevel attention mechanism for motion fatigue recognition based on sEMG and ACC signal fusion
Source: PLoS One. 2024 Nov 4;19(11):e0310035. doi: 10.1371/journal.pone.0310035 (PMC11534257; doi:10.1371/journal.pone.0310035)
Supplement: S1 Table — (DOCX) [file pone.0310035.s001.docx]

Table S 1 Tukey’ HSD of Loss value during training of different classification models

|  | Mean difference | SEM | *q* value | Prob | Sig | Lower limit of the confidence interval | Upper limit of confidence interval. |
| --- | --- | --- | --- | --- | --- | --- | --- |
| Model-2_Loss Model-1_Loss | 0.008 | 0.021 | 0.564 | 0.978 | 0 | -0.046 | 0.063 |
| Model-3_Loss Model-1_Loss | -0.002 | 0.021 | 0.148 | 0.999 | 0 | -0.057 | 0.052 |
| Model-3_Loss Model-2_Loss | -0.010 | 0.021 | 0.713 | 0.957 | 0 | -0.065 | 0.044 |
| Model-4_Loss Model-1_Loss | -0.031 | 0.021 | 2.110 | 0.444 | 0 | -0.086 | 0.023 |
| Model-4_Loss Model-2_Loss | -0.040 | 0.021 | 2.675 | 0.235 | 0 | -0.095 | 0.014 |
| Model-4_Loss Model-3_Loss | -0.029 | 0.021 | 1.961 | 0.508 | 0 | -0.084 | 0.025 |

** At a 0.05 level, the means of the different models are not significantly different.*
