# Supplementary material for: Multilevel attention mechanism for motion fatigue recognition based on sEMG and ACC signal fusion
Source: PLoS One. 2024 Nov 4;19(11):e0310035. doi: 10.1371/journal.pone.0310035 (PMC11534257; doi:10.1371/journal.pone.0310035)
Supplement: S2 Table — (DOCX) [file pone.0310035.s002.docx]

**Table** S 2 Bi-factor variance accuracy analysis of different methods and exercise states.

|  | DF | Sum of squares | Mean square | *F* value | *P* value |
| --- | --- | --- | --- | --- | --- |
| Different methods | 6 | 0.17922 | 0.022 | 126.99 | <0.0001 |
| Different states | 2 | 5.62E-4 | 2.811E-4 | 1.19 | 0.30273 |
| Interaction | 12 | 0.015 | 0.001 | 5.46 | <0.0001 |
| Model | 20 | 0.19 | 0.009 | 41.49 | <0.0001 |
| Error | 1659 | 0.39 | 2.352E-4 |  |  |
| Modified whole | 1679 | 0.58 |  |  |  |

*** *At the 0.05 significance level, it is evident that different methods are significantly different, different states are significantly different, and the interaction between different methods and different states is also significantly different.*
