# Supplementary figures and images for: Multilevel attention mechanism for motion fatigue recognition based on sEMG and ACC signal fusion
Source: PLoS One. 2024 Nov 4;19(11):e0310035. doi: 10.1371/journal.pone.0310035 (PMC11534257; doi:10.1371/journal.pone.0310035)

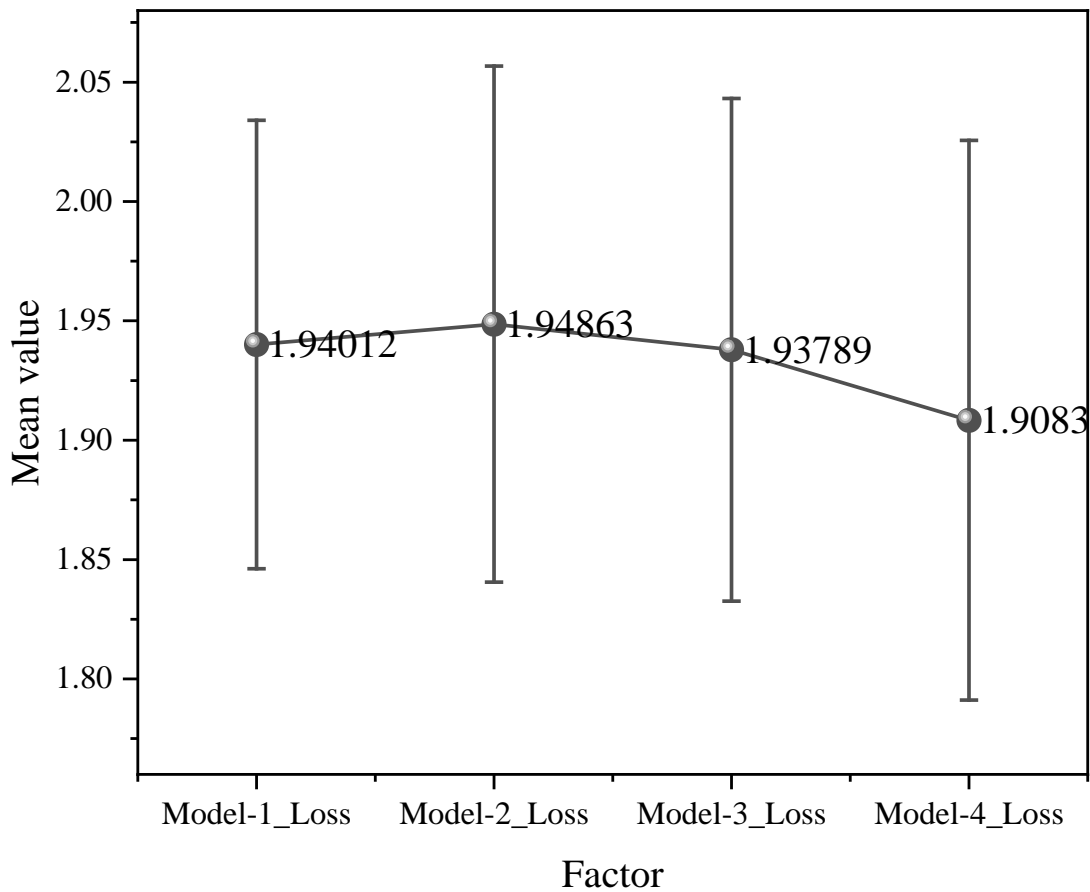

Supplement: S1 Fig — (PDF) [file pone.0310035.s003.pdf]

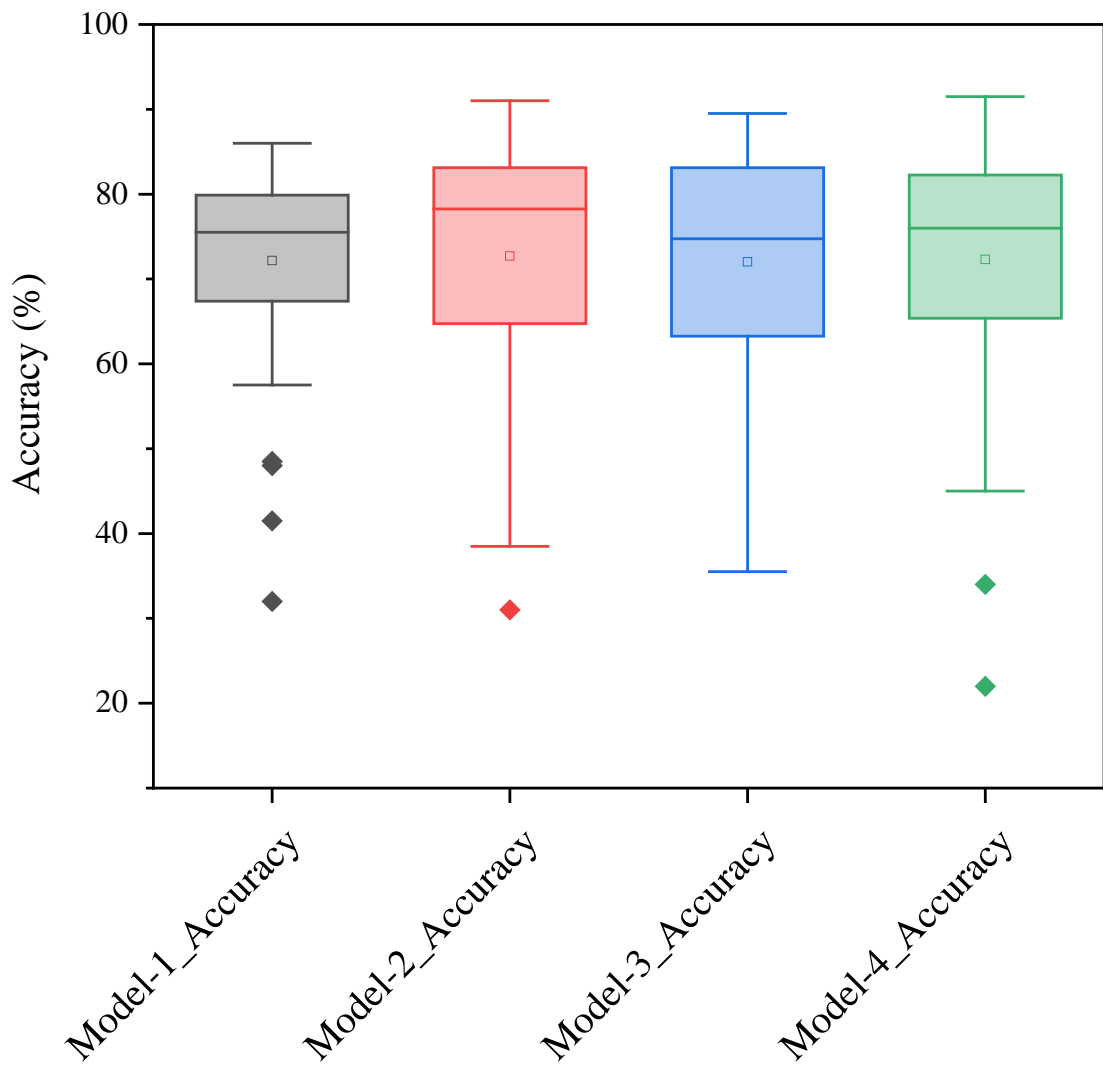

Supplement: S2 Fig — (PDF) [file pone.0310035.s004.pdf]

True label

True label

True label

True label

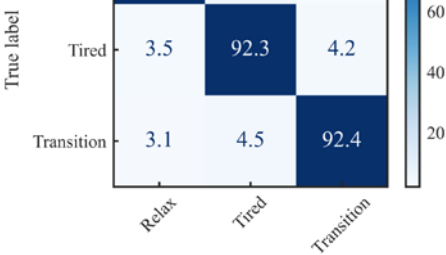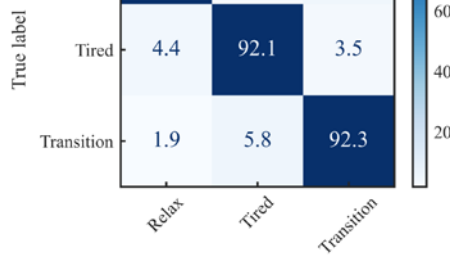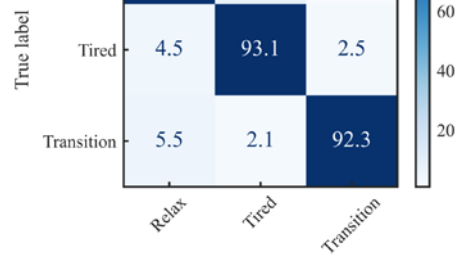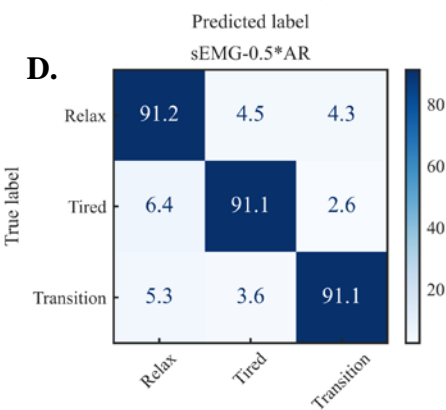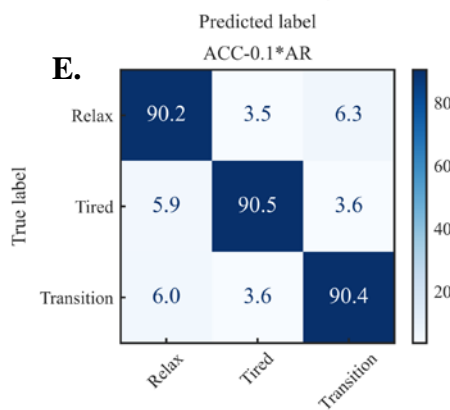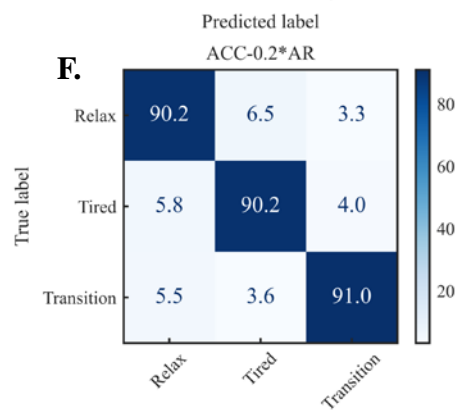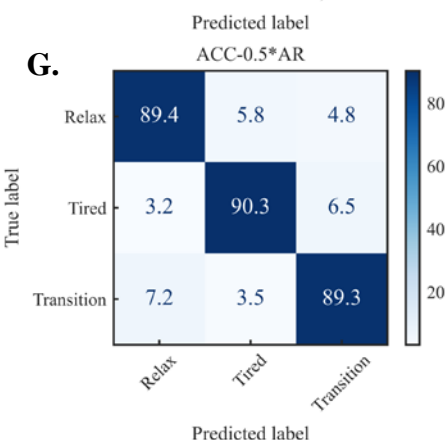

Supplement: S3 Fig — A.sEMG-ACC, B.sEMG-0.1*AR, C.sEMG-0.2*AR, D.sEMG-0.5*AR, E.ACSI-0.1 *AR, F.ACSI-0.2 *AR, G.ACSI-0.5 *AR. (PDF) [file pone.0310035.s005.pdf]

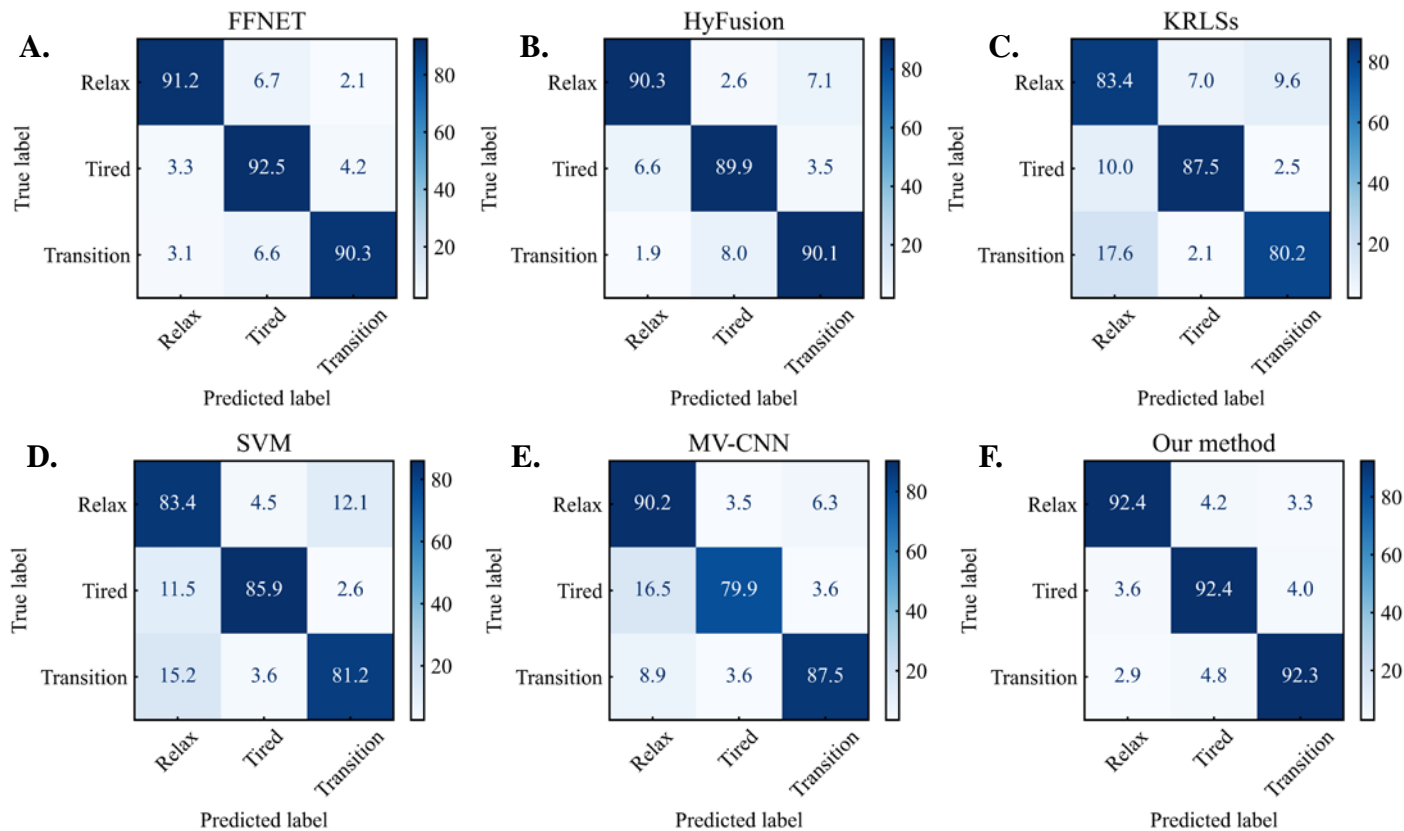

Supplement: S4 Fig — A. FFNET, B. HyFusion, D. KRLSs, E.MV-CNN, F.Our method. (PDF) [file pone.0310035.s006.pdf]
