# Supplementary material for: Multilevel attention mechanism for motion fatigue recognition based on sEMG and ACC signal fusion
Source: PLoS One. 2024 Nov 4;19(11):e0310035. doi: 10.1371/journal.pone.0310035 (PMC11534257; doi:10.1371/journal.pone.0310035)
Supplement: S1 Algorithm — (DOCX) [file pone.0310035.s007.docx]

| Algorithm S 1 Multi-level Attention Mechanism Fusion(MAMF) |
| --- |
| Input: *sEMG, ACC* signals |
| Output: Fused feature vector  Process:  1. Local Feature Attention Mechanism (LFAM)  -Transform sEMG, ACC signals to amplitude envelopes *A*(*t*) using Hilbert transform. |
| - Use *A*(*t*) to calculate attention weights for weighted computation.  2. Dual-scale Attention Mechanism(DSAM)  - Channel Attention Mechanism:  - Perform Global Average Pooling (GAP) on multi-channel data *X* to obtain *x_sq_*.  - Apply activation operation with two fully connected layers to obtain channel weight coefficients *s*.  - Multiply *s* with corresponding channel data for weighted calculation.  **- Neuron Attention Mechanism:**  **-** Calculate neuron energy *e_q_* for each neuron *q* in a channel.  **-** Obtain neuron weight coefficients *w_q_* and *b_q_* through analytical solution.  **-** Perform weighted calculation based on neuron importance.  **3.** **Feature Fusion**  **-** Concatenate the outputs of LFAM and DSAM.  - Apply fullyconnected layer for final fusion.  **Return:** Fused feature vector |
