# Supplementary material for: Multilevel attention mechanism for motion fatigue recognition based on sEMG and ACC signal fusion
Source: PLoS One. 2024 Nov 4;19(11):e0310035. doi: 10.1371/journal.pone.0310035 (PMC11534257; doi:10.1371/journal.pone.0310035)
Supplement: S2 Algorithm — (DOCX) [file pone.0310035.s008.docx]

| Algorithm S 2 Dual-scale Attention Mechanism (DSAM) |
| --- |
| **Input**: Multi-channel data *X*  **Output**: Attention-weighted feature map *Y_r_*  **Process**:  1. Channel Attention Mechanism: |
| - Compress each channel of *X* to obtain channel-wise feature vectors.  -Apply an activation operation (e.g., ReLU) to obtain channel weight coefficients.  -Multiply channel weight coefficients with corresponding channel data.  2. Neuron Attention Mechanism:  - Calculate neuron energy *e_q_* for each neuron *q* in a channel.  - Obtain neuron weight coefficients *w_q_* and *b_q_* through an analytical solution.  - Perform weighted calculation based on neuron importance.  **Return**: Attention-weighted feature map *Y_r_* |
